# Supplementary material for: Interactive analysis of single-cell epigenomic landscapes with ChromSCape
Source: Nat Commun. 2020 Nov 11;11:5702. doi: 10.1038/s41467-020-19542-x (PMC7658988; doi:10.1038/s41467-020-19542-x)
Supplement: Supplementary file 1 — Supplementary Information [file 41467_2020_19542_MOESM1_ESM.pdf]

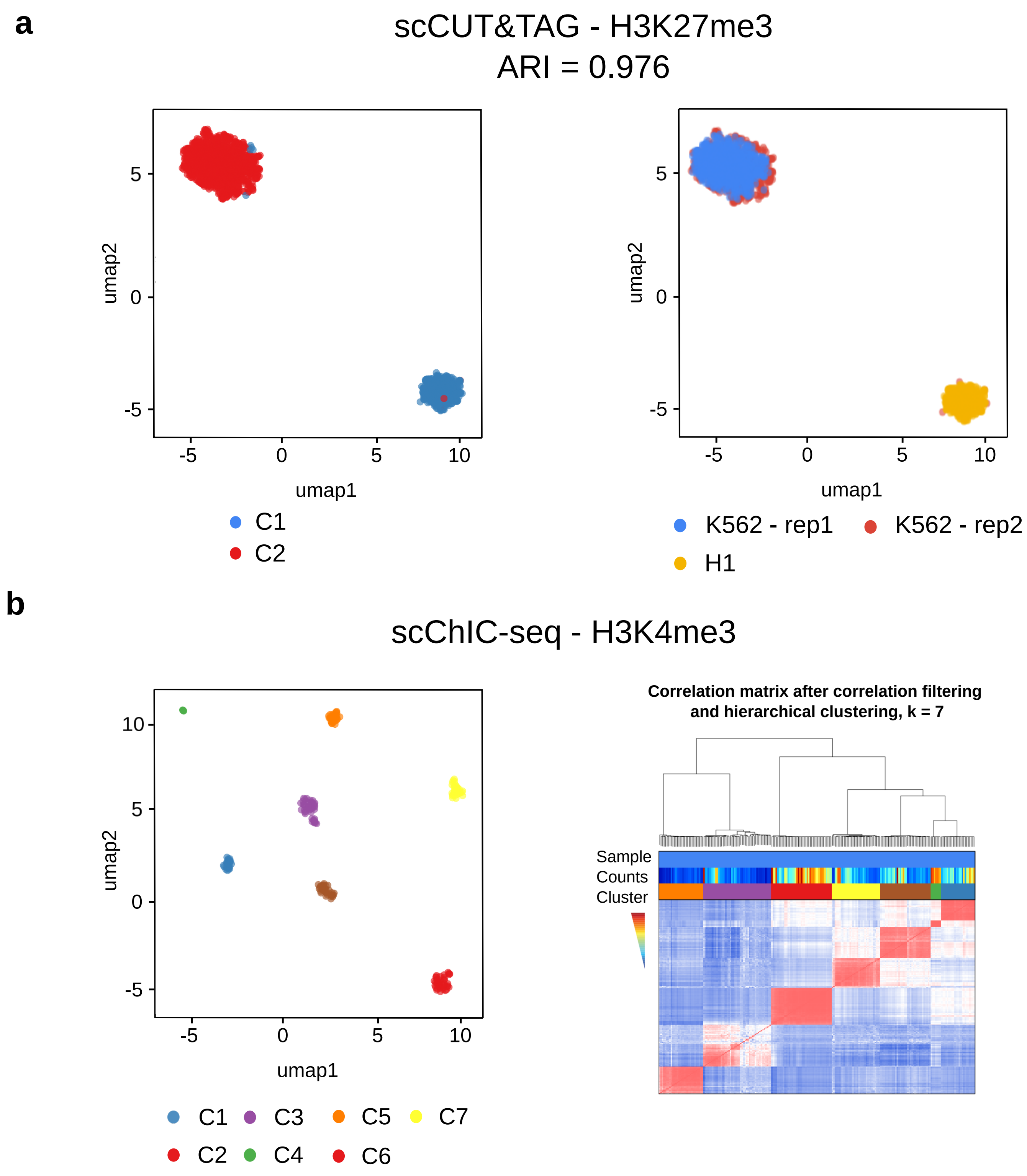

Supplementary Figure 1. ChromSCape identifies subpopulations from various single-cell histone modification profiling technologies. ChromSCape analysis of (a) scCUT&TAG H3K27me3 dataset from Kaya-Okur et al., 2019, comprising two replicates of the K562 cell line and one replicate of H1 cell lines. UMAP plots are colored according to cluster membership (k = 2) and sample of origin, respectively. (b) scChIC-seq H3K4me3 dataset from Ku et al., 2019, comprising one heterogenous human white blood cells. (Left panel) UMAP plot colored according to cluster membership (k = 7); (right panel) Pairwise correlation matrix of Pearson's Correlation scores between cells after correlation filtering.

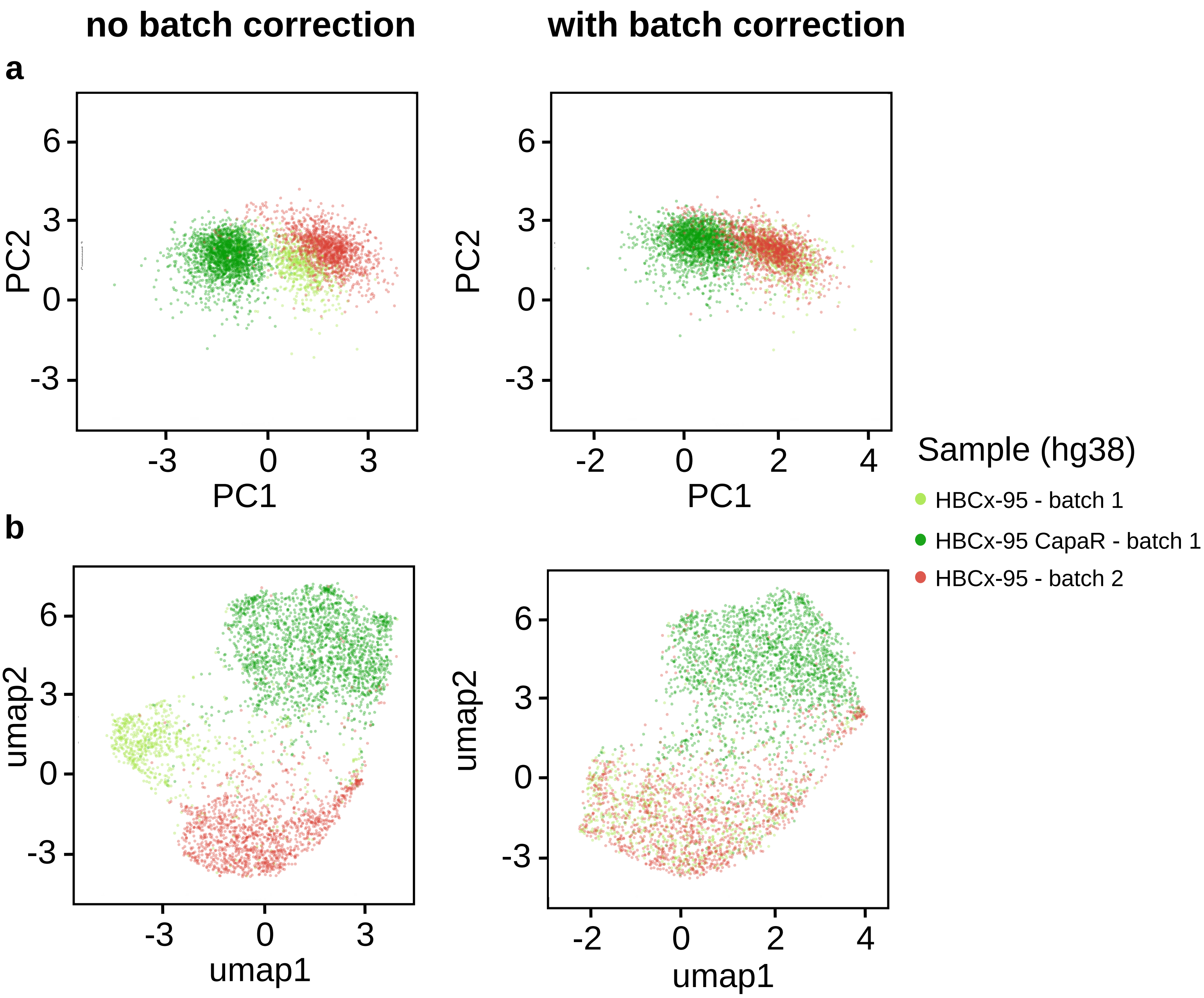

Supplementary Figure 2. Correction of the batch effect between two sets of PDX samples, processed with different batches of hydrogel beads. Samples HBCx-95 and HBCx-95-CapaR PDXs were taken from Grosselin et al., 2019, considered as 'batch 1'; sample HBCx-95-batch 2 is a biological replicate of HBCx95 - batch 1, originating from another mouse, processed with a different batch of beads and our latest data engineering pipeline. ChromSCape was run with default parameters with or without batch correction, PCA (a) and UMAP (b) plots are colored according to sample of origin.

a

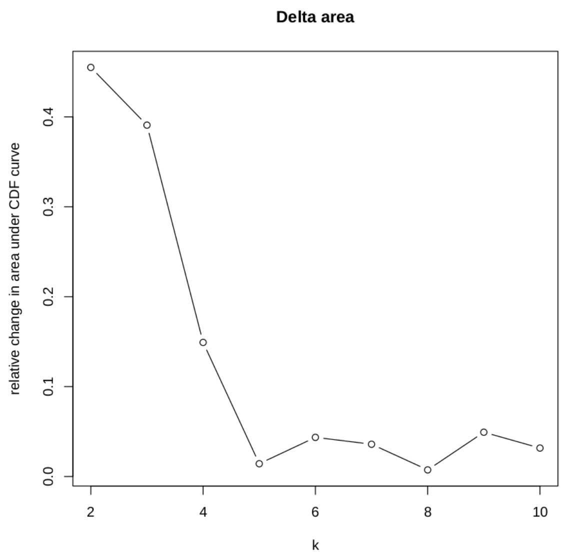

Supplementary Figure 3. Relative change in area under the Cumulative Distribution Fraction for k=2 to 10 clusters. (scATAC-seq dataset (GSE99172) containing 8 cell lines and 4 patient-derived cells) (a) Relative change in area under the Cumulative Distribution Fraction for k=2 to 10 clusters.

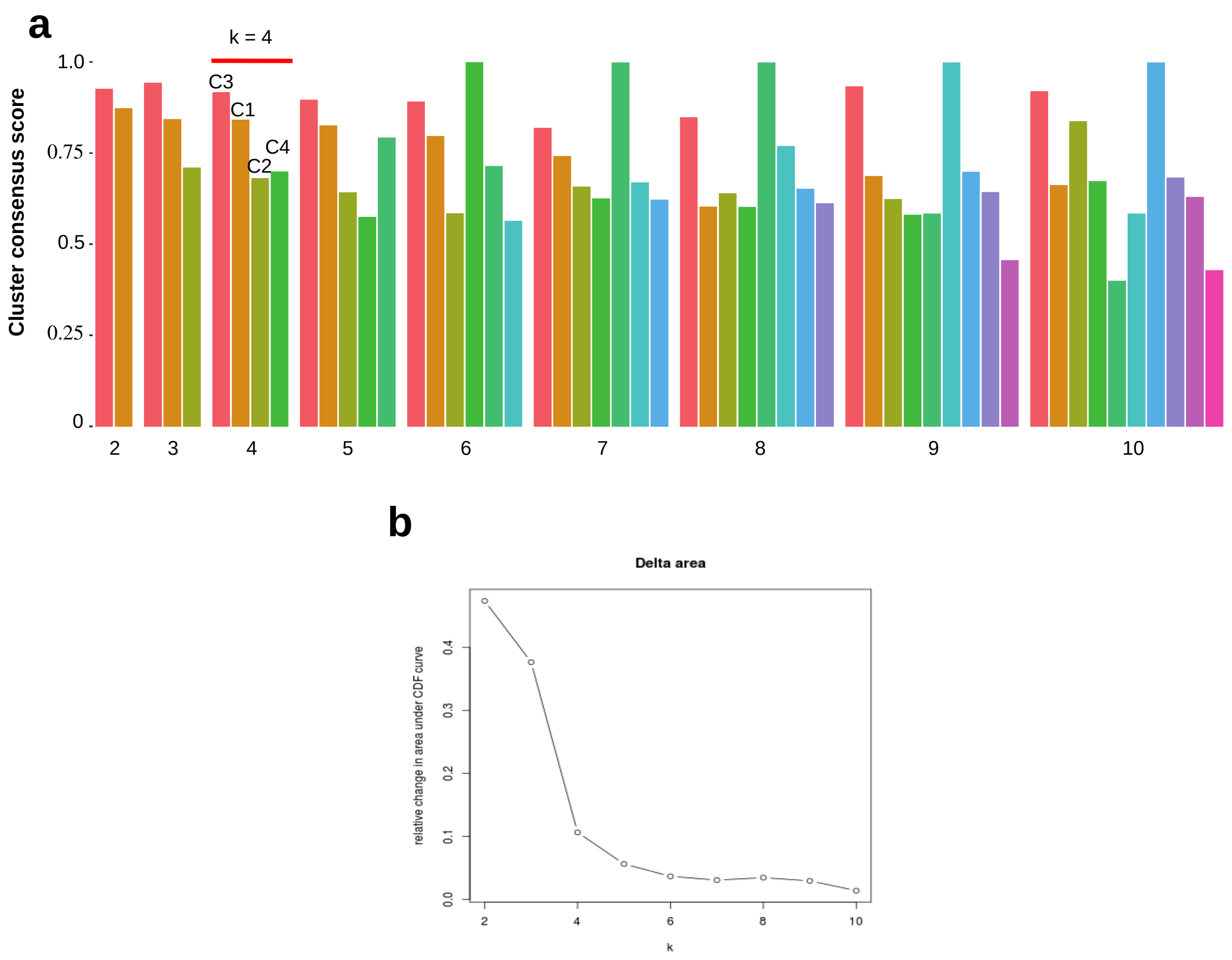

Supplementary Figure 4. Consensus clustering of n=903 mouse stromal cells using 80% of cells at each iteration and 1,000 iterations (samples HBCx-22, HBCx-22-TamR, HBCx-95, HBCx-95-CapaR PDXs). (a) Barplot of consensus scores for each segmentation, from k=2 to 10. k=4 clusters was chosen. The names of the clusters corresponding to the analysis is showed above histogram for k=4. (b) Relative change in area under the Cumulative Distribution Fraction for k=2 to 10 clusters.
