# Supplementary material for: Interactive analysis of single-cell epigenomic landscapes with ChromSCape
Source: Nat Commun. 2020 Nov 11;11:5702. doi: 10.1038/s41467-020-19542-x (PMC7658988; doi:10.1038/s41467-020-19542-x)
Supplement: Supplementary file 3 — Reporting Summary [file 41467_2020_19542_MOESM3_ESM.pdf]

nature research

Corresponding author(s): Céline VALLOT

Last updated by author(s): Sep 15, 2020

Reporting Summary

Nature Research wishes to improve the reproducibility of the work that we publish. This form provides structure for consistency and transparency in reporting. For further information on Nature Research policies, see Authors & Referees and the Editorial Policy Checklist .

Statistics

For all statistical analyses, confirm that the following items are present in the figure legend, table legend, main text, or Methods section.

n/a	Confirmed	
☐	☒	The exact sample size (*n*) for each experimental group/condition, given as a discrete number and unit of measurement
☐	☒	A statement on whether measurements were taken from distinct samples or whether the same sample was measured repeatedly
☐	☒	The statistical test(s) used AND whether they are one- or two-sided<br>*Only common tests should be described solely by name; describe more complex techniques in the Methods section.*
☒	☐	A description of all covariates tested
☐	☒	A description of any assumptions or corrections, such as tests of normality and adjustment for multiple comparisons
☐	☒	A full description of the statistical parameters including central tendency (e.g. means) or other basic estimates (e.g. regression coefficient) AND variation (e.g. standard deviation) or associated estimates of uncertainty (e.g. confidence intervals)
☐	☒	For null hypothesis testing, the test statistic (e.g. *F*, *t*, *r*) with confidence intervals, effect sizes, degrees of freedom and *P* value noted<br>*Give P values as exact values whenever suitable.*
☒	☐	For Bayesian analysis, information on the choice of priors and Markov chain Monte Carlo settings
☒	☐	For hierarchical and complex designs, identification of the appropriate level for tests and full reporting of outcomes
☒	☐	Estimates of effect sizes (e.g. Cohen's *d*, Pearson's *r*), indicating how they were calculated

Our web collection on statistics for biologists contains articles on many of the points above.

Software and code

Policy information about availability of computer code

Data collection	https://github.com/vallotlab/scChIPseq_DataEngineering, including bowtie2 2.2.9 , STAR 2.6.0, bedtools 2.27.1, MACS2 2.1.2, samtools 1.9
Data analysis	Single-cell epigenomics datasets was analyzed with ChromSCape R package, https://github.com/vallotlab/ChromSCape.<br>For benchmarking, codes are available at https://github.com/vallotlab/ChromSCape_benchmarking

For manuscripts utilizing custom algorithms or software that are central to the research but not yet described in published literature, software must be made available to editors/reviewers. We strongly encourage code deposition in a community repository (e.g. GitHub). See the Nature Research guidelines for submitting code & software for further information.

Data

Policy information about availability of data

All manuscripts must include a data availability statement. This statement should provide the following information, where applicable:

- Accession codes, unique identifiers, or web links for publicly available datasets
- A list of figures that have associated raw data
- A description of any restrictions on data availability

H3K27me3 scChIP-seq human in-silico mix of 4 cell types: The samples correspond to n=326 human tumor cells from untreated PDX (HBCx-22), n=201 human T cells (Jurkat) and n=306 B cells (Ramos) taken from 1 and n=454 cells from the MDA-MB-468 triple-negative breast cancer cell line (GSE152502).
H3K4me3 scChIC-seq human white blood cells dataset4: n=285 white blood cells from a human male donor were downloaded as gzipped single-cell BED files from GSE105012, inputted directly into ChromSCape and aggregated into 50kbp bins (default).
H3K27me3 scCUT&Tag human H1 and K562 cells5: A replicate of K562 cell line comprising of n=908 cells from GSE124680, another replicate of n=479 K562 cells and n=486 H1 cells from GSE124690 were downloaded as gzipped single-cell BED files, inputted directly into ChromSCape and aggregated around gene TSS (+/-2500bp).
H3K27me3 scChIP-seq human datasets: The samples correspond to human cells from patient-derived xenograft (PDX) originating from two different human

donors1. For this study, we added a new scChIP-seq dataset, corresponding to a biological replicate of HBCx-95 (GSE152502), processed with a novel batch of hydrogel beads.
scATAC-seq datasets: The scATAC-seq dataset is composed of two cell types derived from two acute myeloid leukaemia (AML) patient (blastocytes (blast) and leukemic stem cells (LSC) from 29) as well as multiple cell lines : GM12878, TF1, BJ, H1, HL60, K562 (3 replicates) from 30, K562 (3 replicates) from 31; monocytes (Mono) and lymphoid primed multipotent progenitor (LMPP) from 29. The count matrix of reads in peaks was downloaded from GEO accession number GSE99172, split into distinct matrices for each sample and formatted to be accepted as input by ChromSCape.
H3K27me3 scChIP-seq mouse datasets: The samples correspond to mouse cells from patient-derived xenograft (PDX) originating from two different human donors1. Raw FASTQ reads were processed using the latest version of our scChIP-seq data engineering pipeline that allowed a more precise removal of PCR and RT duplicates (code available at https://github.com/vallotlab/scChIPseq_DataEngineering) to produce 50kbp binned count matrices given as input to ChromSCape (matrices available at https://figshare.com/projects/Single-Cell_ChIP-seq_of_Mouse_Stromal_Cells_in_PDX_tumour_models_of_resistance/66419).

Field-specific reporting

Please select the one below that is the best fit for your research. If you are not sure, read the appropriate sections before making your selection.

- [x] Life sciences
- [ ] Behavioural & social sciences
- [ ] Ecological, evolutionary & environmental sciences

For a reference copy of the document with all sections, see nature.com/documents/nr-reporting-summary-flat.pdf

Life sciences study design

All studies must disclose on these points even when the disclosure is negative.

Sample size	For PDX experiment, no sample-size calculation was performed. Our objective was to generate a biological replicate of our initial published H3K27me3 scChIP-seq dataset for one animal model (HBCx95) with n>1,000 individual cells.
Data exclusions	We excluded cells from analyses based on pre-established Quality Control metrics, detailed in Methods sections (based on minimal and maximum coverage, and correlation score to other cells). For the scChIP cell mix (4 cell types), as the number of cells in each sample was unbalanced (e.g. the raw MDA-MB-468 containing n=3,382 cells while others have a maximum of n=456 cells), 500 cells from MDA-MB-468 were randomly sub-sampled using ChromSCape 'Perform Subsampling' option. We also removed from the analysis the segments corresponding to known amplifications and homozygous loss of DNA of the Triple Negative Breast Cancer cell line MDA-MB-468, previously found by analyzing the input of bulk ChIP-seq of the same cells, using the BED file containing the identified CNAs (see Supplementary Material).
Replication	For PDX experiment, we generate one biological replicate of our initial published H3K27me3 scChIP-seq dataset for the HBCx-95 model.
Randomization	Randomization is not relevant to our study. For PDX experiments, our objective was to validate a batch-correcting approach for scChIP-seq datasets.
Blinding	No blinding was performed. For PDX experiments, our objective was to validate a batch-correcting approach for scChIP-seq datasets.

Reporting for specific materials, systems and methods

We require information from authors about some types of materials, experimental systems and methods used in many studies. Here, indicate whether each material, system or method listed is relevant to your study. If you are not sure if a list item applies to your research, read the appropriate section before selecting a response.

Materials & experimental systems

n/a	Involved in the study	
☒	☐	Antibodies
☐	☒	Eukaryotic cell lines
☒	☐	Palaeontology
☐	☒	Animals and other organisms
☒	☐	Human research participants
☒	☐	Clinical data

Methods

n/a	Involved in the study	
☒	☐	ChIP-seq
☒	☐	Flow cytometry
☒	☐	MRI-based neuroimaging

Eukaryotic cell lines

Policy information about cell lines

Cell line source(s)	MDA-MB-468 from ATCC HTB-132
Authentication	Human cell lines were obtained from ATCC and not authenticated.
Mycoplasma contamination	The cell lines were negative for mycoplasma contamination.
Commonly misidentified lines (See ICLAC register)	No commonly misidentified cell lines were used in the study.

Animals and other organisms

Policy information about studies involving animals; ARRIVE guidelines recommended for reporting animal research

Laboratory animals	8- to 12-week-old female Swiss nude mice purchased from Charles River and maintained under specific pathogen-free conditions.
Wild animals	The study did not involve wild animals.
Field-collected samples	The study did not involve samples collected from the field.
Ethics oversight	The care and housing of mice used in this study were in accordance with institutional guidelines and the rules of the French Ethics Committee (project authorization no. 02163.02).

Note that full information on the approval of the study protocol must also be provided in the manuscript.
